# Inhibitory effects of inhaled complex traditional Chinese medicine on early and late asthmatic responses induced by ovalbumin in sensitized guinea pigs

**DOI:** 10.1186/1472-6882-11-80

**Published:** 2011-09-24

**Authors:** Hung-Chou Chang, Cheng-Chung Gong, Ji-Liang Chen, Oi-Tong Mak

**Affiliations:** 1 Department of Life Sciences, National Cheng Kung University, Tainan 701, Taiwan; 2 Department of Medical Technology, Chung Hwa University of Medicine Technology, Tainan 717, Taiwan; 3 Office of the Principal, Tainan Tzu-Chi Senior High School, Tainan 708, Taiwan; 4 Department of Healthcare Department, University of Kang Ning, Tainan 709, Taiwan

**Keywords:** asthma, Chinese medicine, guinea pig, nebulization, ovalbumin

## Abstract

**Background:**

Many formulae of traditional Chinese medicines (TCMs) have been used for antiasthma treatment dating back many centuries. There is evidence to suggest that TCMs are effective as a cure for this allergenic disease administered via gastric tubes in animal studies; however, their efficacy, safety and side effects as an asthmatic therapy are still unclear.

**Methods:**

In this study, guinea pigs sensitized with ovalbumin (OVA) were used as an animal model for asthma challenge, and the sensitization of animals by bronchial reactivity to methacholine (Mch) and the IgE concentration in the serum after OVA challenge were estimated. Complex traditional Chinese herbs (CTCM) were administered to the animals by nebulization, and the leukocytes were evaluated from bronchoalveolar lavage fluid (BALF).

**Results:**

The results showed that inhalation of CTCM could abolish the increased lung resistance (13-fold increase) induced by challenge with OVA in the early asthmatic response (EAR), reducing to as low as baseline (1-fold). Moreover, our results indicated higher IgE levels (range, 78-83 ng/ml) in the serum of sensitized guinea pigs than in the unsensitized controls (0.9 ± 0.256 ng/ml). In addition, increased total leukocytes and higher levels of eosinophils and neutrophils were seen 6 hours after challenge, and the increased inflammatory cells were reduced by treatment with CTCM inhalation. The interleukin-5 (IL-5) level in BALF was also reduced by CTCM.

**Conclusion:**

Our findings indicate a novel method of administering traditional Chinese medicines for asthma treatment in an animal model that may be more effective than traditional methods.

## Background

Bronchial asthma is a complex syndrome with many clinical symptoms. It is characterized by variable airflow obstruction, airway hyperresponsiveness and inflammation [1,2]. Provocation of the airway by inhaled allergens is followed by bronchospasm and airway smooth muscle thickening, and accumulation of eosinophils in mucosa and in bronchoalveolar lavage also occurs [1,3]. In addition, according to analyses of bronchial biopsies and lavage samples removed from allergen-exposed asthmatics, contributing to the earlier asthmatic response (EAR) and late asthmatic response (LAR) after allergen challenge [4,5], IgE-dependent activation of mast cells is present with degranulation and subsequent release of several mediators such as histamine, prostanoids and leukotrienes into the lumen of the airway. It is generally agreed that the EAR reaches a peak at about 5-30 min after challenge and lasts for around 2 hours, and in more than 50% of atopic asthmatic patients, the EAR is followed by the LAR, which occurs 4-12 h after challenge and lasts for several hours, even up to a couple of days [6]. The development of allergen-induced airway hyperresponsiveness is correlated with the LAR [7], in which an increase in the quantity of airway inflammatory cells, particularly eosinophils, can be observed in induced sputum [4,6]. A number of small animal models such as mice, rats and guinea pigs have been developed for asthma study, in which the LAR occurs in the airway of sensitized animals after exposure to antigens. Of the animal models, the guinea pig is generally the preferred model because the responses to a variety of contractile and relaxant substances are similar to those of humans [8,9].

For the treatment of asthma, sodium cromoglycate and β_2_-adrenoceptor agonist inhibit the EAR. On the other hand, both the LAR and associated airway hyperresponsiveness are inhibited by sodium cromoglycate and corticosteroids, not by β_2_-adrenoceptor agonists [10-12]. In addition, in most cases, mild-to-moderate asthma can be controlled by inhalational steroids; however, long-term steroid therapy is often associated with multiple side effects that have long-term repercussions on one's health [13]. In contrast to western medicines, Chinese herbs have been used to treat asthma for many centuries, some of which have been proven to inhibit the EAR and LAR in asthma patients subjected to specific allergens [8,14-16]. Freeze-dried powder of a traditional Chinese medicine was administered via a gastric tube before or after challenge in this study. The results showed that the Chinese medicine attenuated the increased respiratory resistance and the amount of eosinophilia induced by the allergens [8,14-16]; however, the mechanisms are unclear. In this study, a formula of complex traditional Chinese medicines (CTCM) was used. This formula contains eight herbs and has been generally used to treat asthmatic patients in Chinese medicine clinics. The CTCM was delivered by inhalation, which we expected to have a better effect than oral administration as the drug can directly and rapidly reach the airways.

In this study, we evaluated the effects of CTCM on the change in the mean pulmonary resistance and the inflammatory responses of the airway in ovalbumin (OVA)-sensitized guinea pigs. The CTCM was nebulized and inhaled autonomically by the animals under anesthesia. The inhalation route is new for CTCM. The effect of local treatment with CTCM on the asthmatic response in sensitized guinea pigs was then investigated.

## Methods

### Sensitization and challenge of animals

The study protocol was approved by the Institutional Review Board of the National Cheng Kung University. Sensitization of the guinea pigs was performed as described in previous studies [17,18] with some modification. Briefly, specific pathogen-free Dunkin-Hartley male guinea pigs (GP) weighing 400-600 g, obtained from the National Laboratory Animal Center, Taipei, Taiwan, were used in this study. The animals were actively IgE-sensitized to OVA (grade VI; Sigma-Aldrich, St. Louis, MO, USA). On day one, 100 μg OVA with 10 mg of aluminum hydroxide (Al(OH)_3_) gel in 0.5 ml of normal saline were injected intraperitoneally. A booster sensitization (50 μg of OVA and 5 mg of aluminum hydroxide gel in 0.25 ml normal saline) was administered on day 7. The sensitized guinea pigs were then exposed to an aerosol containing 1% OVA (w/v) for 20 sec on day 14. Animals were anesthetized with urethane (2 g/kg i.p.), intubated, and challenged by inhalation of nebulized 1% OVA (w/v) for 20 sec on day 21. Unsensitized guinea pigs were treated in the same way with normal saline.

### Preparation of the Chinese herbal formula

In this study, we used a CTCM formula that is used by traditional Chinese medical physicians for the treatment of asthmatic patients. The composition of the CTCM was similar to the xiao-qing-long-tang (XQLT) formula, a formula of Chinese medicine that has been proven to suppress the EAR and LAR in sensitized guinea pigs via oral administration [14]. There are eight common Chinese herbs in this CTCM: Ephedrae herba (stem of Ephedrae sinica Stapf, 18.75 g), Paeoniae radix (root of *Paeoniae lactiflora *Pallas, 18.75 g), Glycyrrhizae radix (root and rhizome of *Glycyrrhiza uralensis *Fischer, 18.75 g), Cinnamonomi ramulus (cortex of *Cinnamomum cassia *Blume, 18.75 g), Asari herba cum radice (whole plant of *Asarum sieboldii *Miq, 18.75 g), bitter apricot seed ( *Prunus armeniaca *(Magnoliaceae), 18.75 g), Common perilla stem (*Perilla frutescens *(Labiatae), 18.75 g), and Ledebouriella root (*Saposhnikovia divaricate *(Apiaceae), 18.75 g). The herbs were purchased from a government-approved herbal company, Hong Cheng Chinese Medicine (Tainan, Taiwan). All air-dried herbs were submerged in 800 ml distilled water for 30 min, then heated to boiling point and maintained at about 103°C for 15 min. The resulting extract was filtered through a 0.45-mm filter and concentrated to approximately 350 ml. The concentrated extract was finally lyophilized to yield 21 g dried powder, which was maintained at 4°C for storage. The dried extract was dissolved in saline before use. For inhalation, three concentrations (0.03, 0.06 and 0.09 g/ml) of CTCM were tested, and the latter two concentrations showed a similar inhibition effect. Therefore, a CTCM concentration of 0.06 g/ml was used in this study.

### The airway hyperresponsiveness (AHR) of Mch

This study examined the dysfunction underlying AHR, including two major physiopathological mechanisms: hypersensitivity and hyperreactivity. Hypersensitivity is a shift to the left of the bronchoconstrictor dose-response curve, and hyperreactivity is an increased slope of the curve. The bronchial hyperresponsiveness of guinea pigs to Mch was recorded to assess the sensitization of the animals. The underlying contributors to airway responsiveness were measured on the basis of the respiratory system resistance to saline and increasing intravenous doses of Mch, as per the method described elsewhere in detail by others [19-21] with some modification. Briefly, to achieve optimal sensitization, we used increasing doses of Mch, at 12.5, 50, 100, 250, and 500 μg/kg.

### Experimental design and administration

Respiratory resistance (**R**_**L**_) was measured before treatment as a baseline control. **R**_**L **_was subsequently recorded after challenge every 5 min in the first hour and every 30 min in the following 5 hours. The guinea pigs were randomly divided into four groups:

1) NSGP (*n *= 10): unsensitized GP challenged with OVA.

2) SGP (*n *= 10): sensitized GP challenged with OVA.

3) MSGP (*n *= 10): sensitized GP treated with 0.06 g/ml CTCM for 5 min by nebulization, followed by OVA challenge (at 5 min after CTCM nebulization).

4) SMGP (*n *= 10): sensitized GP challenged with OVA, followed by treatment with 0.06 g/ml CTCM for 5 min by nebulization (at 10 min after OVA challenge).

### Measurement of airway responses to OVA challenge

Guinea pigs were anesthetized with urethane (2 g/kg; i.p.), tracheostomized, and cannulated with polyethylene tubing (PE210; Scientific Commodities, Lake Havasu City, AZ, USA), then a catheter was advanced through the mouth into the lower end of the esophagus. Pleural pressure was measured via the esophageal catheter. The lateral pressure in the trachea, transpulmonary pressure (the difference between the tracheal and pleural pressure), and R_L _were measured as described in a previous study [22]. A pneumotachograph (Series 1110; Hans Rudolph, Shawnee, KS, USA) connected to the proximal end of the endotracheal tube was used to measure R_L_. The respiratory volume was obtained by digital integration of the flow signal, and R_L _was calculated from the transpulmonary pressure and flow rates at isovolumetric points [22]. Samples of 5-10 breaths were analyzed for each determination of R_L_. In addition, we measured the underlying contributors to airway responsiveness, including airway reactivity (*i.e.*, the slope of the increase in the mean pulmonary flow resistance [R_L_] for a given increase in Mch dose), airway sensitivity (*i.e.*, the lowest dose of Mch that produced bronchoconstriction), and the maximum inducible bronchoconstriction (maximum R_L_). These components of airway responsiveness were measured based on the response of R_L _to saline and increasing intravenous doses of Mch.

### IgE in serum

Blood was withdrawn from the guinea pigs from the heart 6 hr after challenge. The IgE level specific to OVA in the serum was estimated using commercial ELISA kits (MARUKO, Nagoya, Japan).

### Bronchoalveolar lavage

Bronchoalveolar lavage was performed 6 h after OVA challenge. Lung lavage was performed gently using 3 ml of PBS (phosphate-buffered saline) at 37°C. The tracheal cannula was clamped, and the thorax was massaged for 60 s before the bronchoalveolar lavage fluid (BALF) was recovered. This process was repeated once. The recovered lavage samples were cooled on ice and centrifuged at 150 × g for 10 min at 4°C. Approximately 4 mL of fluid were recovered from each sample of BALF, thus maintaining a roughly equal amount of BALF among the treatment groups. The total cell count and cell viability were estimated using a hemacytometer and trypan blue staining. Slides were prepared using a Cytospin model II (Shandon, Pittsburgh, PA, USA). The differential cell count was assessed with Liu's stain. A total of 500 cells were assessed and classified into mononuclear cells, neutrophils, or eosinophils based on the standard morphologic criteria.

### IL-5 in BALF

IL-5 in BALF supernatant was assessed using commercial ELISA kits (R&D Systems, Minneapolis, MN, USA).

### Statistical Analysis

The data from 10 animals in each group were collected, and the results are expressed as mean ± SEM (standard error of the mean). Repeated measures ANOVA or one-way ANOVA was performed to compare the statistical difference between groups. Differences with *P *< 0.05 were judged to be significant.

## Results

### Effects of Mch on the airway

**Figure 1:**
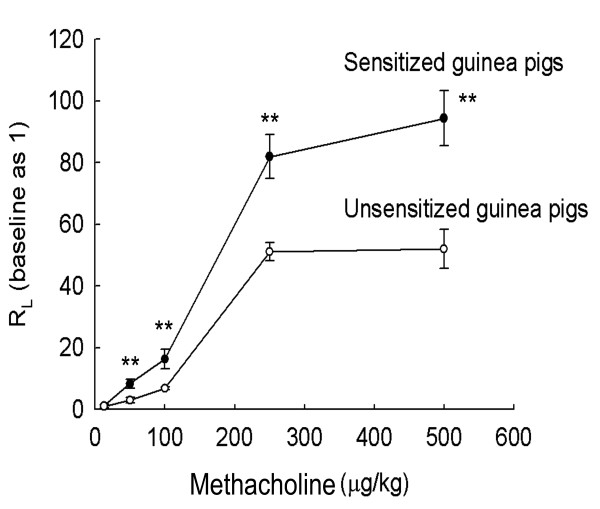
**Airway responsiveness methods. Pulmonary airflow resistance (R**_**L**_**) was measured in response to increasing doses of intravenous methacholine (Mch)**. Using the resulting R_L_-Mch dose-response curve, indices of airway reactivity (Slope R_L_), airway sensitivity, the lowest dose producing bronchoconstriction (Break R_L_) and the maximal degree of bronchoconstriction (Max R_L_) were measured (repeated measures ANOVA with a Mauchly post-hoc test, sensitized vs. unsensitized; **P < 0.01).

The R_L _of the guinea pigs was dependent on the dose of Mch. However, the curve of R_L _in relation to Mch had a greater slope for the sensitized animals than that for the unsensitized animals (Figure 1). Therefore, the results suggested that the sensitized guinea pigs exhibited enhanced airway responsiveness to Mch.

#### IgE in the serum of unsensitized and OVA-sensitized guinea pigs after challenge

**Figure 2:**
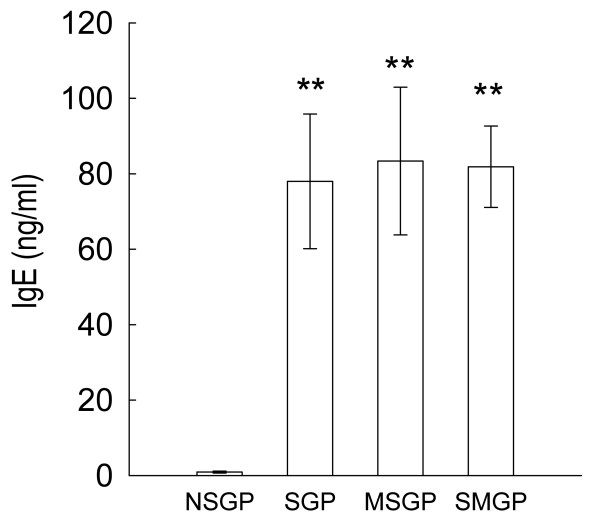
IgE in the serum of guinea pigs. Statistical significance (one-way ANOVA with a Duncan post-hoc test, **P < 0.01) represents a comparison between the sensitized groups (SGP, MSGP and SMGP) and the control group (NSGP). Each column represents the mean of the data from 10 animals and the restical bars represent the standard deviation. NSGP: unsensitized animals with challenge; SGP: sensitized animals with challenge; MSGP: sensitized animals with CTCM treatment 5 min before challenge; SMGP: sensitized animals with CTCM treatment 10 min after challenge.

In the OVA-sensitized GP after challenge (with or without CTCM nebulization), the average OVA-specific IgE in the serum of the sensitized guinea pigs in the SGP group was 78.0 ± 17.83 ng/ml, and the average IgE levels of the MSGP and SMGP groups were about 83.4 ± 19.57 ng/ml and 81.9 ± 10.79 ng/ml, respectively. These values were all significantly higher than the level in the unsensitized control group, which was approximately 0.9 ± 0.256 ng/ml (Figure 2).

### R_L _of guinea pigs after challenge

**Figure 3:**
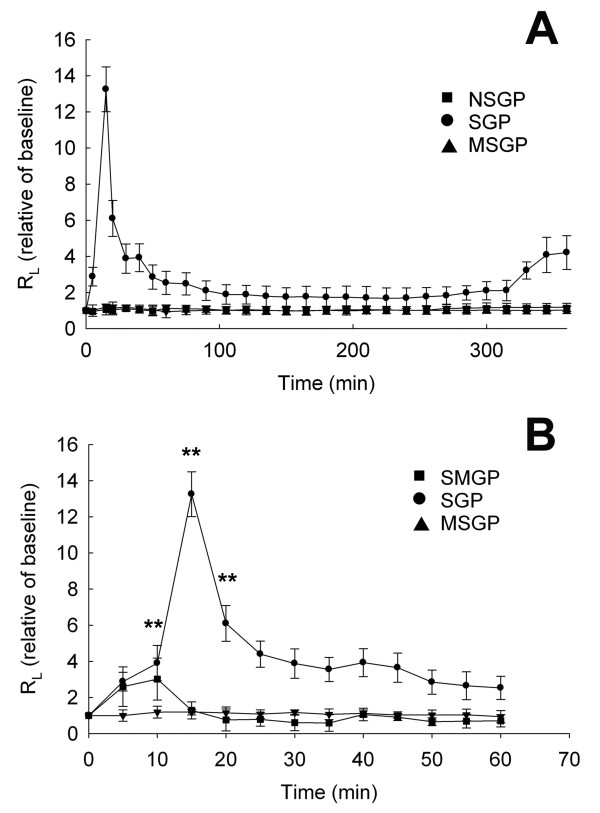
**The effect of CTCM on the change in the respiratory resistance (ratio) after allergen challenge in OVA-sensitized asthmatic guinea pigs**. (A) Overview of the 6-hr period. Comparison between the NSGP, SGP and MSGP groups. (B) Comparison of the different groups of sensitized animals in the first hour (repeated measures ANOVA with a Mauchly post-hoc test, sensitized vs. unsensitized; **P < 0.01). NSGP: unsensitized animals with challenge; SGP: sensitized animals with challenge; MSGP: sensitized animals with CTCM treatment 5 min before challenge; SMGP: sensitized animals with CTCM treatment 10 min after challenge. Each point represents the mean of the data from 10 animals and the restical bars represent standard deviation.

The respirator resistance (**R**_**L**_) was monitored at regular intervals for up to 6 hr after aerosol inhalation of antigens (Figure 3A). Aerosolized OVA caused immediate bronchoconstriction, which peaked at 15 min among the sensitized guinea pigs. In the SGP group, inhalation of OVA increased the R_L _by 13-fold that of the control. In addition, the EAR was also exhibited in the SGP group. The EAR started immediately after OVA challenge and reached a maximum about 15 min after challenge with OVA. At any point in time after challenge, the R_L _in the SGP group was at least 2-fold that in the NSGP group (*P *< 0.01 at 15 min). At around 6 hr after challenge, another increase in R_L _of a moderate level was observed.

### Effect of CTCM on allergen-induced respiratory resistance

For the animals pre-treated with CTCM (the MSGP group), R_L _reduced to the baseline level of the control group after challenge (Figure 3A). The respirator resistance at each time point from 5 min to 1 h after challenge was significantly lower in the MSGP group than in the control SGP group (*P *< 0.01 at 10, 15, 20 min) (Figure 3B). On the other hand, as the maximum increase in R_L _was observed 15 min after challenge, we therefore examined the effect of post-treatment with CTCM 10 min after challenge. Interestingly, the results showed that for the animals post-treated with CTCM (SMGP group), CTCM treatment almost completely impeded the early OVA-induced R_L _response immediately (data not shown in Figure 3A as the line of the SMGP group overlapped with that of the MSGP group). The R_L _of the SMGP group was increased at 5 and 10 min, but the increase in R_L _was attenuated after treatment with CTCM, and R_L _had decreased to the baseline levels of the control and MSGP groups 15 min after challenge (*P *< 0.01 at 15 min; Figure 3B).

### Effect of CTCM on OVA-induced inflammatory cells infiltration in BALF

**Figure 4:**
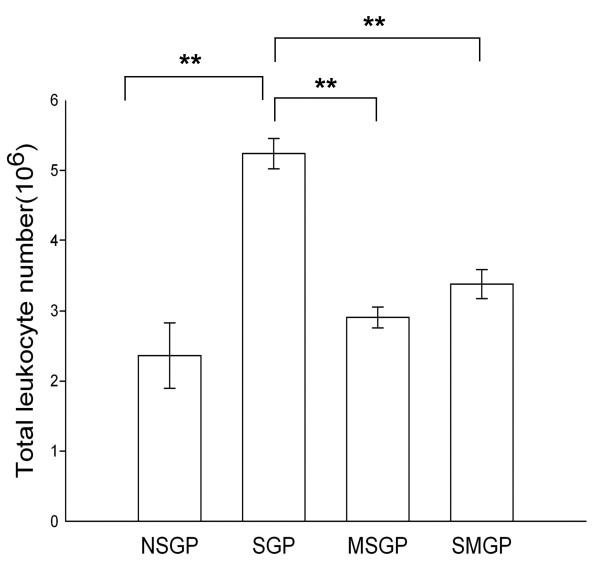
**The effect of CTCM on the change in the total leukocyte cell number 6 hr after allergen challenge in OVA-sensitized asthmatic guinea pigs**. Each column represents the mean of the data from 10 animals and the restical bars represent standard deviation (one-way ANOVA with a Duncan post-hoc test, **P < 0.01). NSGP: unsensitized animals with challenge; SGP: sensitized animals with challenge; MSGP: sensitized animals with CTCM treatment 5 min before challenge; SMGP: sensitized animals with CTCM treatment 10 min after challenge.

**Figure 5:**
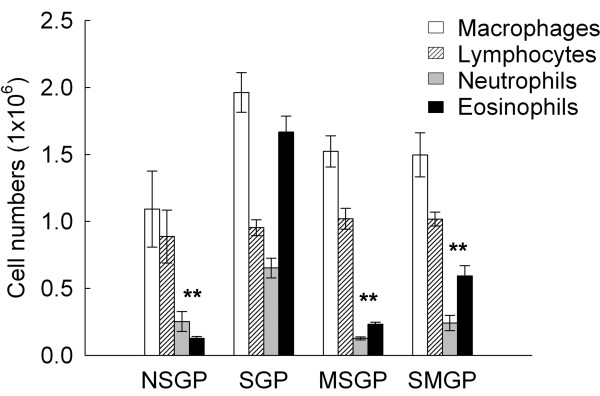
**The effects of CTCM on the changes in differential cell counts after allergen challenge in OVA-sensitized asthmatic guinea pigs**. CTCM treatment before or after OVA challenge decreased the cells numbers of eosinophils and neutrophils (MSGP or SMGP vs. SGP; one-way ANOVA with a Duncan post-hoc test, **P < 0.01). Each group represents the mean of the data from 10 animals and the restical bars represent standard deviation. NSGP: unsensitized animals with challenge; SGP: sensitized animals with challenge; MSGP: sensitized animals with CTCM treatment 5 min before challenge; SMGP: sensitized animals with CTCM treatment 10 min after challenge.

We next recovered inflammatory cells from BALF obtained from the animals at 6 hr. As compared with the non-immunized group, OVA caused a significant increase in inflammatory cells in the immunized group without CTCM treatment (SGP) (Figure 4). There was also a significant increase of eosinophils at this time point after OVA challenge, while CTCM treatment, either before or after OVA challenge, had an inhibitive effect on eosinophil infiltration in BALF, as shown in Figure 5. As compared with the immunized group (SGP), our results showed that CTCM treatment significantly reduced the increase in eosinophils in BALF at 6 h (*P *< 0.01) after challenge. In addition, CTCM treatment also inhibited the increase in neutrophils (*P *< 0.01) in both the CTCM pre- and post-treated groups.

### IL-5 in the BALF of the guinea pigs

**Figure 6:**
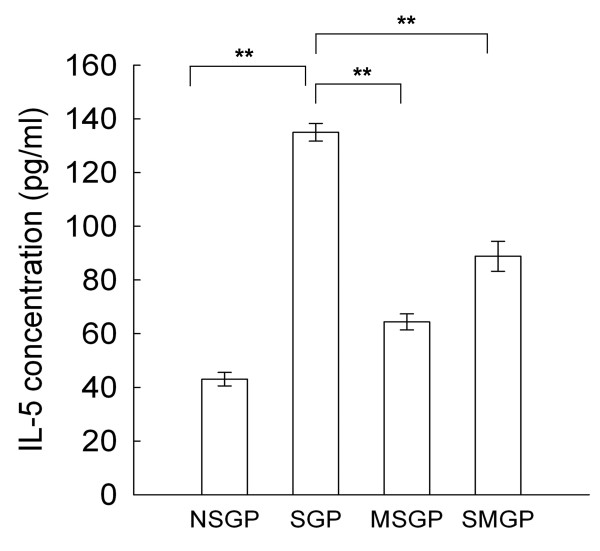
The effect of CTCM on the change in IL-5. CTCM treatment before or after OVA challenge reduced IL-5 levels in the BALF (one-way ANOVA with a Duncan post-hoc test, **P < 0.01: MSGP or SMGP *vs*. SGP). Each group represents the mean of the data from 10 animals and the restical bars represent standard deviation. NSGP: unsensitized animals with challenge; SGP: sensitized animals with challenge; MSGP: sensitized animals with CTCM treatment 5 min before challenge; SMGP: sensitized animals with CTCM treatment 10 min after challenge.

The average IL-5 level in the BALF of the unsensitized guinea pigs (NSGP group) was about 43 pg/ml, and the average IL-5 level of the SGP group was increased by OVA to 135 pg/ml. In the MSGP and SMPG groups, the average IL-5 levels were decreased by CTCM inhalation to 64 pg/ml and 88 pg/ml, respectively (Figure 6).

## Discussion

In this study, we used an OVA-sensitized guinea pig model to investigate the effect of inhalation of CTCM on the early and late antigen-induced airway responses. Guinea pigs have been used as an animal model for asthmatic research for many years. Different sensitization processes may induce different severities of antigen reactions. Booster sensitization by an aerosolized antigen has been widely used in recent years, as this antigen delivery route is similar to the natural physiological way, and this method can elicit responses including elevated levels of allergen-specific IgE, eosinophilic airway inflammation, and AHR [8,14-16]. On the other hand, delivery of medication directly to the airways has a quicker action than the oral route, as the drug can act immediately upon the target area. Therefore, inhalation therapy is generally thought to be the most effective and safe route in comparison with other forms of administration of medications for the treatment of asthma.

Bronchial hyperreactivity to both histamine and Mch has been well established and is used in the clinical diagnosis of asthma [23,24]. Mch is believed to have little or no irritant effect but to act directly on the cholinergic receptors innervated in smooth muscle [25]. In the test of Mch, this nonspecific stimulator induced a larger R_L _in the sensitized subjects than the unsensitized ones. The slope of the dose and R_L _curve in the sensitized groups shifted to the left, as has been observed in other studies [26]. The IgE levels in the serum of the animals in the sensitized groups were also higher than those in the unsensitized animals. These results suggested that the process of sensitization was successful, and that a sensitized model was established. Furthermore, there was no significant difference in the IgE level in the serum between the animals treated with CTCM (MSGP and SMGP groups) and those without CTCM treatment (SGP group). This might be due to the guinea pigs having been sensitized to OVA three times in a total of 21 days; therefore, specific anti-OVA IgE was induced to very high levels. In addition, as the CTCM was applied directly to the airway, it therefore had no significant effect on the level of serum IgE. The results showed that the guinea pigs in the CTCM-treated groups were indeed sensitized and that bronchoconstriction was abolished by CTCM.

Chinese herbs have been used for the treatment of asthmatic patients for centuries. Most of the Chinese herbs used for the treatment of bronchial asthma are either administered orally or through a gastric tube for research purposes. For example, several studies have demonstrated the anti-asthmatic effects of traditional Chinese medicine formulas such as XQLT by administering the medicine via the gastric route to guinea pigs [14-16], and simplified herbal medicine treatment also reduced several asthma features in a mouse model [27,28]. Currently, many of the western medicines used for the treatment of asthma are inhaled. Although several CTCM formulas have been shown to relieve asthma symptoms by oral administration, no study has been found in the English literature in which CTCM was administered in inhaled form to validate its efficacy. Administration of CTCM by inhalation, such as the use of portable bronchodilator sprays, which are already widely used in asthma treatment, is convenient and relatively simple. Inhalation it is expected to have a better effect than oral administration, as the CTCM can directly and rapidly reach the airways. For practical application, it is possible to use an aerosol inhaler to deliver CTCM into the lungs.

In this study, we demonstrated that CTCM administered in nebulized form through the intratracheal route is capable of inhibiting both the early and late asthmatic responses and the influx of inflammatory cells into the airways of guinea pigs following bronchial antigen challenge. The results indicate that this CTCM is a bronchoprotective substance and exerts an anti-inflammatory effect in OVA-sensitized guinea pigs. This is the first time that Chinese medicines have been used in the treatment of bronchial asthma by inhalation in an animal model. The EAR and LAR to OVA challenge in guinea pigs were observed to be dramatically inhibited by CTCM treatment in the present study.

When anti-asthmatic drugs are administered prior to antigen challenge, sodium cromoglycate inhibits both the EAR and LAR, whereas both the LAR and associated airway hyperresponsiveness are inhibited by sodium cromoglycate and corticosteroids [29]. Albuterol, a β_2_-agonist, blocks the EAR but neither inhibits the LAR nor affects eosinophil infiltration [29]. Dexamethasone inhibits the LAR, but not the EAR, and inhibits the increase in eosinophils in BALF 24 hr after antigen challenge [30]. In this study, the effects of CTCM treatment on antigen-induced airway responses were investigated, and our results showed inhibition of the increased R_L _and eosinophilia by CTCM. We have demonstrated that nebulized CTCM exerts both a bronchodilator and anti-inflammatory activity in OVA-sensitized guinea pigs. Inhalation of CTCM both 5 min before challenge (MSGP group) and 10 min after challenge (SMGP group) significantly inhibited the EAR and the increase of inflammatory cells in the airways, including eosinophils and neutrophils. These findings suggest that CTCM can be used as an anti-asthmatic agent and is useful for the prevention or treatment of asthma.

Many compounds extracted from herbs have demonstrated anti-oxidant properties and anti-inflammatory effects, which can lead to a decrease in allergic tissue injury. For example, green tea extract has been reported to have an anti-inflammatory activity that inhibits iNOS expression by suppressing IFN-γ-elicited STAT-1 activation [31], and aqueous extract of *Arbutus unedo *L. has been shown to attenuate carrageenan-induced lung inflammatory injury, which is associated with the inhibition of IL-6 production, reduction of STAT-1 and STAT-3 expression, and downregulation of iNOS and ICAM-1 mRNA expression [32]. It is still unclear whether the CTCM used in this study has a similar mechanism to the mechanisms observed for other herbal extracts that possess inhibition properties to transcription factors or have direct effects on pro-inflammatory genes. This is worthy of further investigation to explore the mechanisms involved.

Many studies have shown that development of the LAR is associated with recruitment of eosinophils into the airway, which presumably cause cellular activation and bronchoconstriction [33-36]. These reactions are due to the release of cytotoxic granular-associated proteins by eosinophils, including major basic protein, eosinophil cationic protein, and eosinophil peroxidase, which have a profound effect on the airway [16,37,38]. Furthermore, eosinophil activation results in the release of other mediators, such as leukotriene C_4 _and platelet-activating factor, which can cause contraction of the airway smooth muscles [39,40]. In the present study, an increase in eosinophils in response to OVA challenge was observed, and the administration of CTCM inhibited the eosinophilia in OVA-sensitized guinea pigs. IL-5 is an important cytokine for eosinophil development and activation, and secretion of IL-5 causes eosinophil recruitment into the airways during the allergic airway response [41,42]. The results of this study showed that IL-5 in BALF was induced by OVA, and that the CTCM could reduce the increased IL-5.

Although the presence of eosinophils in airway inflammation is recognized as an important event, it does not fully explain the inflammation observed in asthma. Increased numbers of neutrophils have been found in patients with acute or persistent asthma compared with controls [43,44]. Many studies have also reported that increased neutrophil levels may contribute to exacerbation in asthma patients exposed to allergens, and airway neutrophils may be associated with asthma severity [45]. Although it is still controversial, there is increasing evidence to support the hypothesis that neutrophils are important in the pathophysiology of asthma. Our results also demonstrated that CTCM inhibited the OVA-induced increase of neutrophils, which suggests that CTCM inhalation might block neutrophil recreation to the site of the allergic reaction.

## Conclusion

In conclusion, we have demonstrated for the first time that nebulized CTCM is effective against early-phase airflow obstruction in anaesthetized sensitized guinea pigs challenged by OVA inhalation. The OVA-induced increases in airway eosinophils and neutrophils and the increased levels of IL-5 in BALF in the late phase were also inhibited by nebulized CTCM treatment. Our results suggest that there may be two anti-asthmatic mechanisms for CTCM, a bronchodilator effect resulting from its stimulation of β_2_-receptors on bronchial smooth muscles, and an ability to inhibit the increased IL-5 and eosinophil infiltration into the airway. The precise mechanism of nebulized CTCM in asthma remains to be elucidated, but the effect of reducing early and late antigen-induced airway responses highlights a possible novel way of administering CTCM for asthma treatment.

## Competing interests

The authors declare that they have no competing interests.

## Authors' contributions

HCC conceived and designed the study and carried out many of the culture experiments, analyzed and interpreted the data, and drafted the manuscript. CCG and JLC performed some of the experiments and data analysis, and contributed to the drafting of the manuscript. OTM was involved in the conception and design of the study and the supervision of experiments. All authors read the manuscript, contributed to its correction, and approved the final version.

## Pre-publication history

The pre-publication history for this paper can be accessed here:

http://www.biomedcentral.com/1472-6882/11/80/prepub
